# Biopharmaceutical Development of a Bifonazole Multiple Emulsion for Enhanced Epidermal Delivery

**DOI:** 10.3390/pharmaceutics11020066

**Published:** 2019-02-02

**Authors:** Joaquim Suñer-Carbó, Ana Calpena-Campmany, Lyda Halbaut-Bellowa, Beatriz Clares-Naveros, María José Rodriguez-Lagunas, Elena Barbolini, Joanna Zamarbide-Losada, Antonio Boix-Montañés

**Affiliations:** 1 Department of Pharmacy and Pharmaceutical Technology and Physical Chemistry, Faculty of Pharmacy and Food Sciences, University of Barcelona, Av. Joan XXIII s/n, 08028 Barcelona, Spain; jsuner@ub.edu (J.S.-C.); anacalpena@ub.edu (A.C.-C.); halbaut@ub.edu (L.H.-B.); elena.barbolini@libero.it (E.B.); joannazamarbide@gmail.com (J.Z.-L.); 2 Institute of Nanoscience and Nanotechnology (IN2UB), University of Barcelona, Diagonal 645, 08028 Barcelona, Spain; beatrizclares@ugr.es; 3 Department of Pharmacy and Pharmaceutical Technology, Faculty of Pharmacy, University of Granada, Campus de la Cartuja, s/n. 18071 Granada, Spain; 4 Department of Biochemistry and Physiology, Faculty of Pharmacy and Food Sciences, University of Barcelona, Av. Joan XXIII s/n, 08028 Barcelona, Spain; mjrodriguez@ub.edu

**Keywords:** multiple emulsion, bifonazole, pseudoplastic, drug release, skin penetration, skin permeation, skin integrity, TEWL, epidermal histology

## Abstract

Efficient topical delivery of imidazolic antifungals faces the challenge of overcoming its limited water solubility and its required long-lasting duration of treatments. In this paper, a hydrophilic multiple emulsion (ME) of Bifonazole (BFZ) is shown to maximize its skin retention, minimize its skin permeation, and maintain an acceptable level of being harmless in vivo. The formulations were pharmaceutically characterized and application properties were assessed based on viscosity measurements. Non-Newtonian pseudoplastic shear thinning with apparent thixotropy was observed, facilitating the formulation retention over the skin. The in vitro release profile with vertical diffusion cells showed a predominant square-root release kinetic suggesting an infinite dose depletion from the formulation. Ex vivo human skin permeation and penetration was additionally evaluated. Respective skin permeation was lower than values obtained with a commercial O/W formulation. The combination of amphoteric and non-ionic surfactants increased the bifonazole epidermal accumulation by a factor of twenty. This fact makes the possibility of increasing its current 24 h administration frequency more likely. Eventual alterations of skin integrity caused by the formulations were examined with epidermal histological analysis and in vivo preclinical measurements of skin elasticity and water retrograde permeation. Histological analysis demonstrated that the multiple emulsions were harmless. Additionally, modifications of in vivo skin integrity descriptors were considered as negligible.

## 1. Introduction

Bifonazole [1-[[1,1’-biphenyl)-4-phenylmethyl]-1*H*-imidazole) (BFZ) is an imidazolic antifungal drug indicated against skin or mucosal mycoses. Its topical administration requires an intensive drug retention in skin because the pathogenicity of dermatomycoses is related to their ability to gain access to other target tissues [1,2]. Formulations for local therapy are desirable in order to achieve the highest drug penetration deep into the epidermis, which is the site of action. Nevertheless, these drugs are markedly lipophilic and are largely retained in the stratum corneum (SCR) [3], making the treatment of deep-seated epidermal infections more difficult. Topical agents, which are conventionally used for the treatment of skin fungal infections, are usually formulated as creams, lotions, or gels. Several formulation strategies, such as the hydrophilic W/O/W multiple emulsions (ME), can expedite this skin accumulation: They consist of two hydrophilic internal and external phases separated by a lipophilic layer that dissolves hydrophobic drugs and incorporates them into a hydrophilic formulation.

Multiple emulsions have attracted considerable attention in recent years due to their application as potentials systems for drug delivery. In this case, they have been investigated as potential skin delivery systems for bifonazole. Many authors [4] describe MEs as “emulsions of emulsions” since they are formed by phase inversion of a simple emulsion. Thus, at least two surfactants are required: One with low HLB (Hydrophilic Lipophilic Balance) forms the primary W/O emulsion and another surfactant with a high HLB achieves the secondary o/w emulsification. In this sense, polymeric emulsifiers provide a strong, thick, and well-defined film around the W/O interface [5]. Additionally, polysorbates (O/W emulsifiers) and sorbitan esters (W/O emulsifiers) are non-ionic surfactants that enhance the formulation stability and flexibility, and furthermore, they facilitate the skin penetration of the drug [6]. They are stable in acids, do not react with ionic ingredients or actives, and have a history of being safe in their use [7]. In summary, polymeric surfactants in combination with conventional small molecular weight emulsifiers, are suitable emulsifiers for multiple emulsions [8] and are additionally useful to enhance the solubilization of poorly soluble drugs [9].

General advantages of W/O/W multiple emulsions are the selective protection of the entrapped drug, their ability to incorporate different actives in the different emulsion phases and their sustained release effects [10]. They have been widely applied in various fields of pharmacy [11,12], such as vehicles for anticancer agents [13] or prolonged drug delivery systems [14]. In Bifonazole formulations, MEs are more suitable than other similar disperse systems, such as liposomes or micelles: The internal phase of liposomes is hydrophilic and is not able to dissolve significant amounts of lipophilic drugs [15,16] such as Bifonazole, whereas the intermediate lipophilic phase of the ME allows to achieve greater solubilization.

The aim of this study was to evaluate the efficiency of nonionic co-emulsifiers in the outer aqueous phase of a multiple emulsion of Bifonazole for topical application to achieve a lower BFZ human skin permeation and larger skin retention in comparison with commercial O/W formulation. In aggregate, the feasibility of an enhanced (that is lengthened) interval of administration, which maintains an acceptable skin tolerance, has been investigated.

## 2. Materials and Methods

W/O/W emulsions were prepared with a two-step emulsification method and were physically characterized with optical examination, laser and microscopic droplet size distribution, rheological studies, pH and conductimetric analyses, and they were tested for drug content. In vitro release and ex vivo human skin permeation were also investigated. After a histologic evaluation of its harmlessness, the in vivo skin integrity of the different formulations, including a commercial emulsion (BFZ-CF) was confirmed after their acute administration in humans.

### 2.1. Substances and Reagents

Bifonazole (BFZ, CAS 60628-96-8), sodium chloride, cetyl palmitate (CP), and polysorbate 80 (Tween^®^ 80, HLB 15) were obtained from Acofarma (Barcelona, Spain). Sorbitan stearate (Span^®^ 60, HLB 4.7) was obtained from Fagron (Terrassa, Spain). The lipophilic surfactant cetyl dimethicone copolyol (Abil^®^ EM90, HLB 5, CDC), an amphoteric surfactant cocamidopropyl betaine (Tego^®^ Betain F, CMB), and the thickening acrylic carbomer (Tego^®^ Carbomer 341ER, TGC) were supplied by Evonik (Essen, Germany). Capric/caprylic triglyceride (Labrafac^®^ Lipophile 1349, LP), were supplied by Gattefossé (Saint-Priest, France). Deionized water (Ω^−1^ < 0.07 mS/cm) used in all experiments was produced by ourselves in-house in a Milli-Q^®^ system (Millipore Iberica S.A.U., Madrid, Spain). Methanol HPLC was obtained from Fisher Scientific (Madrid, Spain). Ammonium acetate, ethanol, sodium dodecyl sulphate, sodium hydroxide, and potassium monohydrogen phosphate K_2_HPO_4_ were obtained from Panreac (Barcelona, Spain). Hank’s balanced salt solution was obtained from Thermo Fisher Scientific (Barcelona, Spain). Reagents for histological preparations were purchased from Sigma and Thermo Fisher Scientific (Barcelona, Spain). Commercial formulation of 1% Bifonazole from Bayer AG (Canesmycospor^®^, BFZ-CF) was used as a comparable formulation.

### 2.2. Preparation of Multiple Emulsions

Two, previously optimized, W_1_/O/W_2_ multiple emulsions with insoluble imidazolic antifungals [17] were prepared with a two-step emulsification method [18]. Compositions are summarized in Table 1.

Primary W_1_/O emulsion was prepared by slow addition of the aqueous phase containing the electrolyte (NaCl) to the oil phase containing BFZ (1%, *w*/*w*) at 80 ± 2 °C under continuous stirring at 250 rpm until approximately 25 °C. The oil phase was prepared dissolving BFZ in a combination of drug solvent and cosolvent (LP and CP) aided with the lipophilic emulsifying agents (CDC and sorbitan stearate) at 80 ± 2 °C. In the second stage, the obtained primary emulsions were slowly added to the corresponding outer aqueous phases (W_2_) 50:50 under 250 rpm at room temperature. After complete addition of the primary W_1_/O emulsion over the external gelified aqueous phase, the resulting mix was paddle stirred for a further 10 min until a homogeneous W_1_/O/W_2_ multiple emulsion had been completely formed.

External aqueous phases (W_2_) had been prepared previously by dispersing the cross-linked TGC polymer, in a co-solvent system of deionized water and the hydrophilic emulsifying agents (CMB and/without polysorbate 80) were neutralized with 10% NaOH solution (10%, *w*/*v*) to obtain a pH value of 6.5–7.0.

### 2.3. Physicochemical Characterization and Stability

Organoleptic characteristics were monitored to detect any visible signs of instability such as creaming, phase separation, or color changes. Droplet homogeneity and size distribution were investigated by optical microscopy (400×) with a Leica DM 1000 LED light microscope (Leica Microsystems, Wetzlar, Germany). Measurements were performed directly and at 1:20 water dilution, just after formulation and monthly during a six-month stability storage period. Droplet size was also analyzed by laser diffractometry with a Malvern Mastersizer 2000 (Malvern Instruments, Worcestershire, UK). The relative distribution in size ranges was calculated based on Mie theory and reported as D[*v*,0.1], D[*v*, 0.5], and D[*v*,0.9], i.e., standard percentile readings of the distribution. The volume-weighted mean diameter, D[4,3] and the volume-surface mean diameter, D[3,2], were also obtained [19]. They were measured (*n* = 3, water-dispersed, room temperature) at 24 h and then at 180 days after preparation. Conductivities (μS/cm) of the 1:20 diluted W_1_/O/W_2_ emulsions were measured (*n* = 3) with a Crison GLP 30 (Crison Instruments, Alella, Spain) at 24 h and 180 days after preparation. pH was measured (Crison Instruments, Alella, Spain) at the same time (*n* = 3, room temperature). All results were compared for statistical significance with Student’s t-test (*p* < 0.05).

The occurrence of destabilization phenomena was assessed by multiple light scattering with a Turbiscan^®^ Lab Expert (Formulaction, L’Union, France). Diluted samples (~35 mL) were placed into cylindrical glass measuring cells, and were completely scanned for light scattering by a reading head that consists of a pulsed near-infrared light source (λ = 880 nm) and two synchronous optical detectors. A pattern of the backscattered light as a function of the sample height was obtained giving a macroscopic fingerprint of the sample at each predetermined time. Measurements (*n* = 3) were performed at 24 h after preparation and after six months storage at room temperature. Thermal stability tests were also performed under different storage conditions for both primary and multiple emulsions. Test tubes were stored vertically and kept at 4 ± 0.1 °C (in refrigerator), 30 ± 0.1 °C, 40 ± 0.1 °C in stability cabins (Heraeus B5042 E and I 42, Madrid, Spain) and room temperature. Observations were made each week during storage.

### 2.4. Rheological Properties

Skin application properties (apparent thixotropy and elasticity) were characterised with rotational and oscillatory tests. Rheological measurements were performed with a Haake Rheostress 1^®^ rheometer (Thermo Fisher Scientific, Karlsruhe, Germany) connected to a thermostatic circulator Thermo Haake Phoenix II + Haake C25P. Data were analyzed with Haake Rheowin^®^ Data Manager v. 3.3 software (Thermo Electron Corporation, Karlsruhe, Germany).

#### 2.4.1. Rotational Measurements

Steady-state measurements were addressed with a cone and a mobile upper cone (C60/2° Ti:60 mm diameter, 2° angle). The shear stress (*τ*) was measured as a function of the shear rate (*γ*). Viscosity curves (*η* = f(*γ*)) and flow curves (*τ* = f(*γ*)) were recorded at 25 ± 0.1 °C. The shear rate ramp program included a 3 min ramp-up period from 0 to 100 s^−1^, 1 min constant shear rate period at 100 s^−1^, and 3 min ramp-down from 100 to 0 s^−1^. Representative mathematical models were fitted to flow curves (when they were non-Newtonian) searching for the best descriptive model: Bingham, Ostwald de Waele, Herschel-Bulkley, Casson, and Cross [20]. Selection of the best fitting was based on the correlation coefficient (observed vs. predicted) and chi-square value. The apparent thixotropy (Pa/s) was estimated as the area of hysteresis loop. Steady-state viscosity (*η*, Pa s) was determined from the constant shear section at 100 s^−1^ and also from the ramp-up period at 10 s^−1^.

#### 2.4.2. Dynamic Oscillatory Measurements

Oscillatory tests were performed with a parallel plate-and-plate geometry (Haake PP60 Ti, 60 mm diameter, 0.5 mm gap) using the same rheometer described above. Firstly, the oscillatory stress sweep test was performed at a constant frequency of 1 s^−1^ from 0.01 to 100 Pa to determine the linear viscoelastic region (LVR). Afterwards, frequency sweep test was carried out between a 0.01 and 10 s^−1^, at a constant shear rate within the LVR. This was in order to determine the related variations of the storage modulus (G′), loss modulus (G″), phase angle (*δ*), and the complex viscosity (η*). During each sweep stress test, the changes in storage and loss modulus and the phase angle (G’, G’’ and δ) were plotted as a function of shear stress at a frequency of 1 Hz.

### 2.5. Drug Content

#### 2.5.1. Drug Extraction

Formulation amounts equivalent to about 5 mg BFZ were mixed with 5 mL of methanol and dispersed in an ultrasound bath for 10 min. Afterwards, the sample was centrifuged at 3000 rpm/10 min and the resulting clear solution was transferred to a vial. After appropriate dilutions with methanol/ pH 7.4 buffer phosphates (75:25), the resulting solution was filtered through 0.45 µm nylon disposable filter (Teknokroma, Barcelona) and measured spectrophotometrically for bifonazole content.

#### 2.5.2. UV Analysis

BFZ concentrations in assay formulations and release samples (see Section 2.6) were measured by UV-spectrophotometry [21] at *λ* = 254 nm (Thermo^®^ Spectronic Helios Beta, Thermo, Cambridge, UK). Calibration curves were prepared with methanol:pH 7.4 phosphates buffer 75:25 (*v*/*v*). Validation according to standard guidelines [22] demonstrated an adequate intraassay and interassay precision (maximum RSD +4.36%) and accuracy (from −3.12 to +2.58%). Linearity ranged between 2.61 and 10.00 μg/mL. The statistically significant determination coefficients (*R*^2^) were obtained on each run as a linearity indicator on each run. The recovery in formulation assays was tested in triplicate using a 1% BFZ methanolic solution (MethSol), giving a result of 93.4% (RSD 7.6%) and this was considered acceptable for our purposes.

#### 2.5.3. HPLC Analysis

BFZ concentration in skin permeation samples (see Section 2.7) was quantified with a Waters Alliance 2695 HPLC (Waters, Milford, MA, USA) with a C18 column Brisa LC2, 5μm, 4.6 mm × 250 mm (Teknokroma, Barcelona, Spain). The mobile phase was methanol:ammonium acetate 65 mM (65:35, pH 3.6) [23] at a flow rate of 1 mL/min at 25 °C. Detection was monitored at 220 nm from 1.56 to 100.00 μg/mL. Accuracy was lower than +3.7%. As for the precision, maximum RSD was 0.8% with an acceptable linearity falling in the calibration range. Statistically significant determination coefficients (*R*^2^) were obtained on each run as a linearity indicator on each run.

#### 2.5.4. Statistical Analyses

Descriptive statistics, confidence intervals and regressions were calculated with the Graphpad^®^ Prism software v.3 (Graphpad Software, San Diego, CA, USA).

### 2.6. In Vitro Drug Release

In vitro BFZ drug release was tested from 300 mg donor amounts of each of the following, ME, BFZ-CF and MethSol with 2.54 cm^2^ vertical diffusion cells [24] using 0.45µm mesh nylon membrane (Teknokroma, Barcelona, Spain). The receptor solution was a well-stirred solution of 12 mL Methanol:K_2_HPO_4_ Buffer 0.05M 75:25 (*v*/*v*) at 32 ± 0.5 °C. Samples from the receptor compartment (300 µL) were taken during a minimum of 5 h with immediate spectrophotometric analysis and blank reposition. Comparative release profiles were obtained (*n* = 3) for each formulation.

#### 2.6.1. Drug Release Equations

Zero order, first order, Hyperbola, Weibull, and Higuchi equations [25] were fitted to the cumulative released BFZ amounts using a non-linear regression routine with Graphpad^®^ Prism 3 software (Graphpad Software, San Diego, CA, USA).

The best model was selected based on the minimum value of the Akaike information criterion (AIC) [26] calculated as follows from the residual sum of squares obtained with each regression equation:

AIC = *n*·ln SSQ + 2*P*, (1)

where SSQ is the residual sum of squares and *P* the number of parameters. Best fitting was considered to be as having the minimum AIC value. Descriptive release parameters were: Release rate *K* (of the best function), released percentages at five hours (%Q5), and efficiency, described as:

Efficiency = AUC_0_^5^/(Q5·*T*), (2)

where AUC_0_^5^ is the individual area under the curve of released amounts and *T* is the time for the first asymptotic value [27].

#### 2.6.2. Statistical Analyses

Comparisons of parameters were performed by one-way ANOVA with GraphPad^®^ Prism 3 software (Graphpad Software, Sant Diego, CA, USA).

### 2.7. Drug Permeation—Penetration

BFZ human skin permeation from each formulation was investigated with 0.64 cm^2^ vertical diffusion cells [24]. Permeation flux and skin retention were calculated for each replicate (*n* = 3).

Skin was obtained from leftovers of the abdominal region of a 38-year old healthy woman during plastic surgery (Hospital de Barcelona, SCIAS, Barcelona, Spain) following a donation procedure of biologic residues approved by Bioethics Committee of the Barcelona-SCIAS Hospital (reference number: BEC/001/16). The patient provided written informed consent. After chirurgical excision, skin was immediately debrided, immersed in Hanks’ solution, and stored at −20 °C. Prior to the experiment, skin laminar samples (400 μm) were obtained with an electrical dermatome (Aesculap GA 630, Tuttlingen, Germany). Skin specimens were mounted on diffusion cells and a receptor compartment was filled up with Ethanol:Transcutol^®^ P:Water (50:20:30 *v*/*v*/*v*). After temperature equilibration at 32 °C, the skin integrity was verified measuring its transepidermal water loss (TEWL) (Tewameter*^®^* TM300, Köln, Germany) just before formulation application (300 mg) and discarding results falling out of the range 4 to 8 g/h/m^2^ [28].

#### 2.7.1. Drug Permeation

At predefined times, 300 μL sample were taken with replacement during a minimum of 32 h. Permeated BFZ amounts per unit area (Q_t_) were obtained by HPLC-UV as described previously. Permeation extent was described with the last time amount (Q32). Permeation flux (J) was calculated by linear regression between Q_t_ and time in the linear phase.

#### 2.7.2. Drug Penetration

At the end of each experiment, the residual amount of donor formulation was withdrawn and the skin specimens were rinsed with aqueous 0.5% sodium laurylsulphate and later deionized water. Resulting skin specimens were blotted dry, weighed, and minced. Drug extraction was run with fresh receptor solution under ultrasounds (×3). Collected solutions were centrifuged (1500 rpm, 10 min) and immediately analyzed. Drug retention levels were reported as the ratio between the amount of drug in skin and the weight of skin sample (µg/g), and as the amount of drug per surface unit (µg/cm^2^).

#### 2.7.3. Statistical Analyses

Statistical comparisons (α = 0.05) were performed by one-way ANOVA using the GraphPad^®^ Prism 3 software (Graphpad Software, San Diego, CA, USA).

### 2.8. Epidermal Histology

Histology of epidermal specimens after the skin permeation-penetration experiments (see Section 2.7) was investigated with optical microscopy. The formulations evaluated were: BFZ-CF, JMLP01BT and MEs placebos (without BFZ). After the permeation experiments, residual contents of donor formulations were mechanically withdrawn and skin samples were set overnight in 4% buffered formaldehyde at room temperature and embedded in paraffin, cut into 5 µm thick sections, stained with hematoxylin-eosin and finally they were observed at ×400 with an Olympus BX41 microscope and Olympus XC50 camera (Olympus, Tokyo, Japan).

### 2.9. Skin Integrity Assessment

Eventual formulation-induced variations of skin integrity were investigated with simple non-invasive in vivo measurements of skin elasticity, stratum corneum hydration (SCH), and transepidermal water loss (TEWL) [28] in an investigator-blinded parallel clinical study.

The study was based on previous knowledge [29] and conducted in accordance with the Declaration of Helsinki [30] and the Ethics Committee on human experimentation of the University of Barcelona (Reference number: iRB00003099).

#### 2.9.1. Subjects

Ten caucasian male or female volunteers, aging from 20 to 52 years old were randomly entered in the study. After being fully informed, each volunteer signed a document stating they had been informed of their agreement, declaring they had understood the study design, the potential risks, and that they were taking part in the study of their own free will. The volunteers agreed to refrain from using body care cosmetics or moisturizers on the flexor side of the left forearm for one week prior to the measurements. Known previous irritation episodes or allergies to any type of topical formulations, soaps, or surfactants were considered a reason for exclusion. The exclusion criteria were: (a) Three days before the study, having used cosmetics for skin care, humectants, etc., on the arms, (b) known previous skin irritation episodes or allergies to any type of topical formulation (pharmaceutical or cosmetical), and (c) virgin TEWL values outside 4 to 8 g/h/m^2^ were discarded.

#### 2.9.2. Test Procedure

The individuals stayed in the test room at least 20 min prior to the measurements (room conditions 22 ± 3 °C and 55% relative humidity). Their skin temperature was measured with a Skin Thermometer^®^ ST500 (Courage and Khazaka, Electronic GmbH, Köln, Germany). Circles of 7 cm in diameter were drawn on the left volar forearm of the volunteer, which is the skin area for the application of each formulation. Then, a measurement of each parameter was taken (*t*_0_). Afterwards, the formulations were applied in the middle of each circle by means of a soft circular movement with the thumb. One hour after the application of the formulations (*t*_1_), a similar set of measurements was obtained at the same skin area. The effects were expressed as mean values of individual differences (*t*_1_–*t*_0_) of each parameter and formulation.

#### 2.9.3. Skin Parameters

The following parameters were tested:

- **Skin elasticity**. The effect of the formulation on the elasticity of the upper skin layers was tested with a Cutometer^®^ MPA 580 (Courage and Khazaka, Electronic GmbH, Köln, Germany). This measurement generates a negative pressure, drawing the skin into a probe that leads to a vertical deformation. When the negative pressure is switched off, the skin recovery is characterized [31,32] in terms of skin biomechanical properties.
- **Corneum stratum hydration** (SCH). It was performed with a Corneometer^®^ 825 (Courage and Khazaka, Electronic GmbH, Köln, Germany). This measures the capacitance variation of the dielectric properties of epidermic *stratum corneum* due to changes in skin hydration.
- **Transepidermal Water Loss** (TEWL). The retrograde water permeation through skin was measured with a Tewameter^®^ TM 300 (Courage and Khazaka, Electronic GmbH, Köln, Germany). This measures the vapor density gradient across the skin combining temperature and relative humidity sensors located in a hollow cylinder applied on the skin surface.

#### 2.9.4. Statistical Analyses

Individual differences were analyzed with a one-way Anova and a Bonferroni post-test to impute the differences, using graphpad Prism^®^ V. 5.00 (GraphPad Software Inc., San Diego, CA, USA).

## 3. Results

### 3.1. Physicochemical Properties and Stability

The prepared simple W_1_/O emulsions appeared as white, odorless, greasy, homogeneous and with a high consistency. The W_1_/O/W_2_ multiple emulsions appeared as white, odorless and homogenous without any signs of precipitation of drug, phase separation, creaming, or any visual change of appearance in any of the samples kept at 5 and 30 °C. At 40 °C, MEs became unstable after 10 weeks storage.

**Figure 1:**
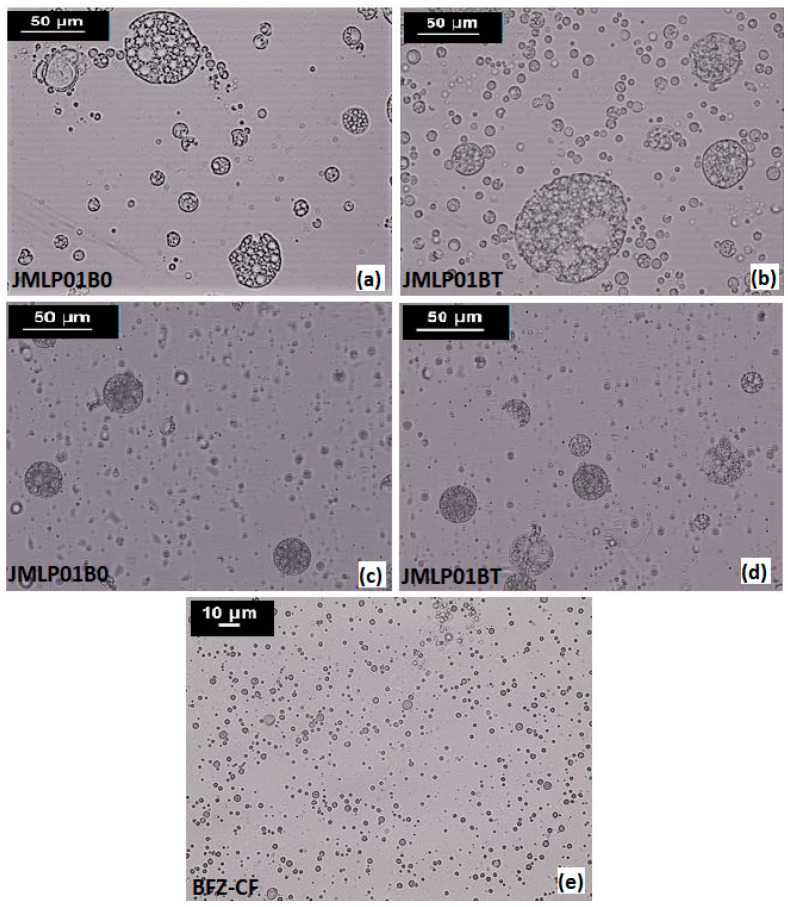
Optical photomicrographs at 400× magnification of JMLP01B0 (**a**,**c**), JMLP01BT (**b**,**d**) and BFZ-CF (**e**) during the storage period (**a**,**b**: At 24 h of preparation; and **c**,**d**: At six months of preparation).

The multi-compartmental structure of both MEs was confirmed by optical microscopy. Multiglobules containing many small droplets in the internal phase are shown in Figure 1. Internal droplets (primary W_1_/O emulsion) ranged 1 to 5 μm. The diameters of multi-globules ranged between 10–50 μm and were later confirmed by laser diffractometry. An optical microphotograph of BFZ-CF is available in Figure 1. Droplets of O/W BFZ-CF ranged about 5 μm, similarly to the primary W_1_/O emulsion of the MEs.

Concerning pH and conductivity, MEs remained stable during six months until 30 °C. Conductivities at 24 h and after six months are summarized in Table 2. Statistically significant differences (*p* < 0.05) were found between the results of both MEs, but not for their differences between zero and six months. No significant pH variations were detected along the stability study.

Laser diffractometry confirmed the results of optical microscopy. Droplet size (D[4,3]) resulted to be 65.2 ± 0.8, 68.5 ± 1.0 and 29.8 ± 9.8µm for JMLP01B0, JMLP01BT, and BFZ-CF, respectively, as also detailed in Table 2.

**Figure 2:**
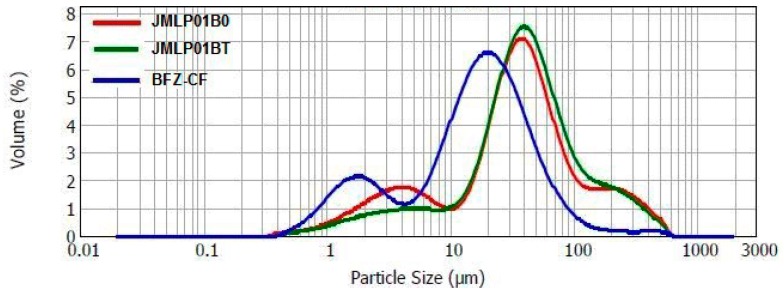
Droplet size distribution for JMLP01B0, JMLP01BT, and BFZ-CF as volume percentages.

A combined plot of particle size distributions of JMLP01B0, JMLP01BT (after 24 h of preparation) and BFZ-CF is shown in Figure 2.

**Figure 3:**
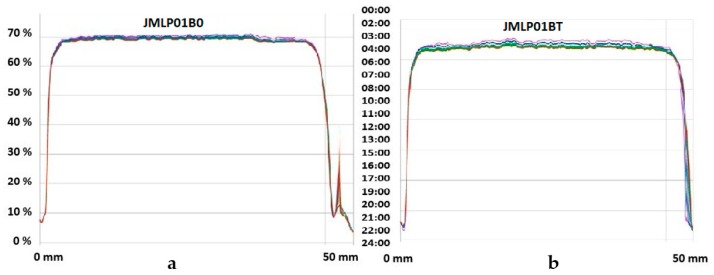
Backscattering profiles (%∆BS, left ordinates) recorded at room temperature of JMLP01B0 (**a**) and JMLP01BT (**b**) at different times (0 to 24 h, right ordinates). Sample cell length (0 to 50 mm) is shown on abscissa.

The long-term physical stability of the MEs, predicted with the Turbiscan Lab^®^ Expert, used only backscattered light (BS); this was because the formulations were not transparent. No relevant variations of the droplet volume fraction (migration) or mean size (coalescence) were observed (Figure 3).

### 3.2. Rheological Properties

#### 3.2.1. Rotational Test

**Figure 4:**
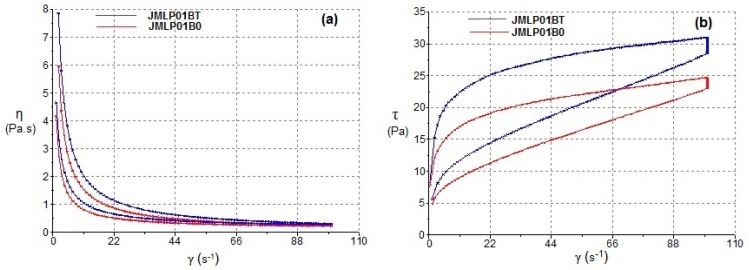
Viscosity (**a**) and flow (**b**) curves of MEs as functions of shear rate after 24 h of preparation.

The results of steady-state rheology as a function of shear rate are summarized in Table 2. The formulations exhibited non-Newtonian pseudoplastic flow and shear thinning behavior with a consistent decrease in viscosity with increasing shear rate from 0 to 100 s^−1^. The Cross model showed the best fitting to both ascending and descending stretches. The flow curves (Figure 4) indicated certain apparent thixotropy as the rheograms displayed a moderate hysteresis loop with the downward curve below the upward curve.

**Figure 5:**
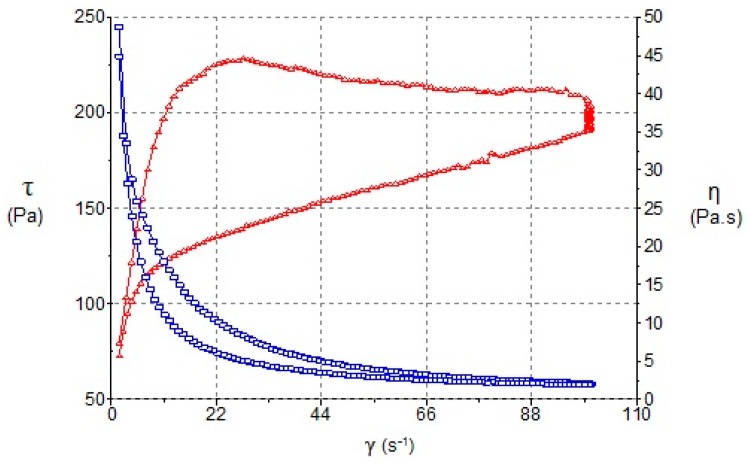
Viscosity (blue line) and Flow curve (red line) of BFZ-CF as a function of shear rate.

Viscosity of BFZ-CF (at 10 and 100 s^−1^) was markedly higher than MEs values (Table 3 and Figure 5).

#### 3.2.2. Oscillatory Test

**Figure 6:**
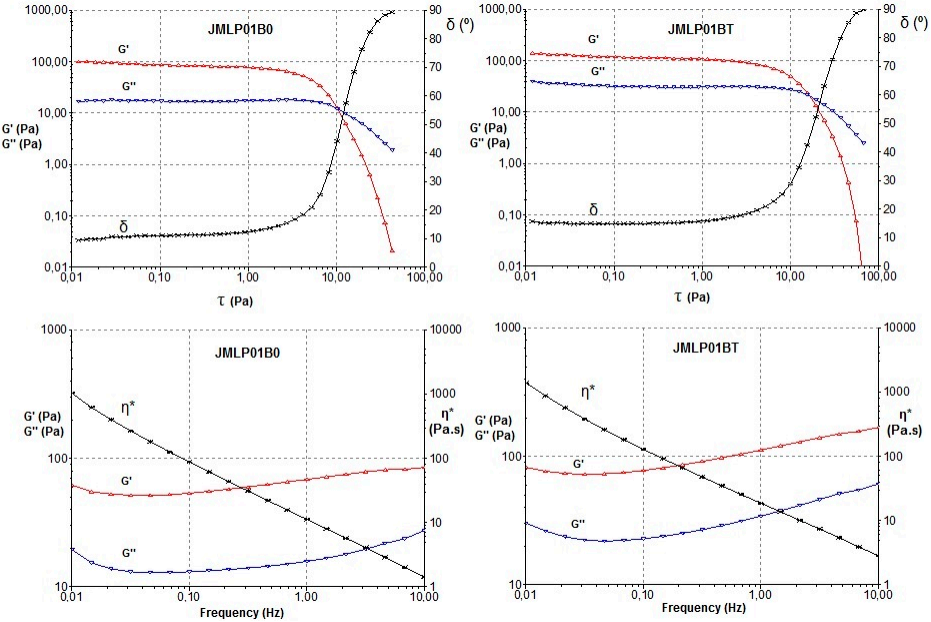
Storage modulus (G’), loss modulus (G’’) and phase angle (δ) of JMLP01B0 and JMLP01BT during a sweep stress test (top). Storage modulus (G’), loss modulus (G’’) and complex viscosity (η*) versus frequency during a frequency sweep test for JMLP01B0 and JMLP01BT (bottom) at 24 h of preparation.

Measurements were performed to find the critical stress. As shown in Figure 6, the critical stress was found at stresses below approximately 2 Pa. Following on from these results, a constant shear stress of 1 Pa (50% of the critical value) was selected to perform the frequency sweep tests. Results for both ME formulations revealed, in this frequency range, a prevalence of the elastic over the viscous behavior (G’>G’’), indicated in what is known as the “elastic plateau” and is shown in Table 3 and Figure 6. Additionally, a higher viscosity was observed for JMLP01BT, confirming previous rotational results.

Throughout the stability study, viscosity tended to be lower at end-times (see Table 3). The differences were not statistically significant (*p* > 0.05). All the cases were at room temperature.

### 3.3. Drug Content

Formulations JMLP01B0 and JMLP01BT (after 30 days of preparation) contained 90.6 ± 1.8% and 95.8 ± 2.2%, respectively, of the nominal drug content in the oil phase. BFZ-CF contained 94.1 ± 1.7% of the declared content of BFZ (mean, SD).

### 3.4. Release Test

**Figure 7:**
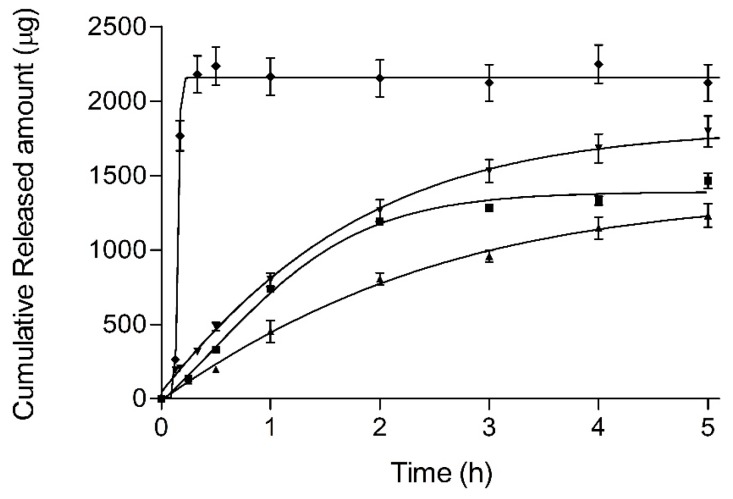
Release profiles (mean values and standard deviations) of BFZ (squares JMP01BT, triangles up JMP01B0, triangles down BFZ-CF, rhombus 1% methanolic solution).

Figure 7 outlines the BFZ release profiles (mean and SD) for each formulation and a methanolic solution reference. After 5 h, mean percentages (%Q5) were 41.0% and 48.8% for JMLP01B0 and JMLP01BT (asymptotic), respectively.

Based on AIC values, individual profiles were best described with the Higuchi equation in all cases except for the methanolic solution (Weibull). Higuchi release parameters are summarized in Table 4. In the case of the methanolic reference, the sigmoidicity of Weibull function is *β* = 9.6 ± 5.5 with a td 0.161 ± 0.009 h, as expected from a liquid solution. Based on ANOVA test, Bonferroni’s multiple comparison tests showed differences between JMLP01B0 and the BFZ-CF (*p* < 0.05). In other cases, the highest efficiency [27] was achieved with the ME containing polysorbate, although differences were not statistically significant between the three formulations (*p* > 0.05).

### 3.5. Skin Permeation

**Figure 8:**
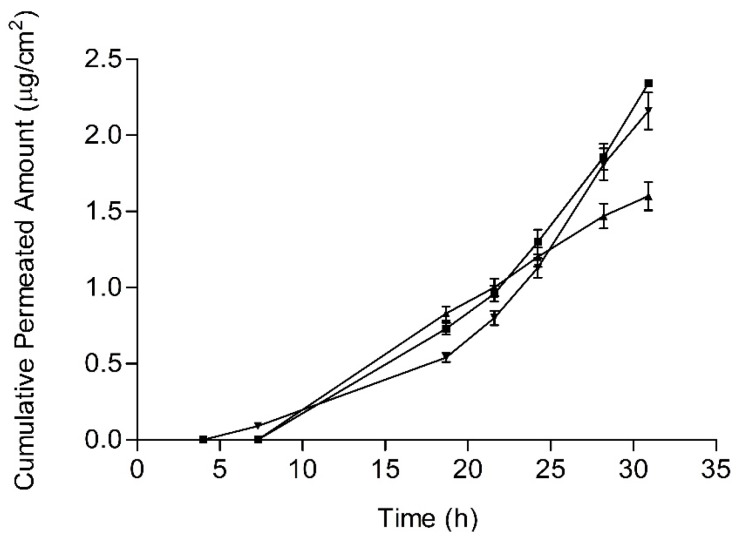
BFZ human skin permeated amounts (mean values and standard deviations) for the BFZ-CF (squares), JMLP01B0 (triangles down) and JMLP01BT (triangles up).

Permeation parameters and penetration values are summarized in Table 3. Statistically relevant differences (*p* < 0.05) were found between JMLP01BT and the other two formulations. The mean skin permeation profiles of BFZ of the three formulations are plotted in Figure 8.

### 3.6. Histological Analysis

**Figure 9:**
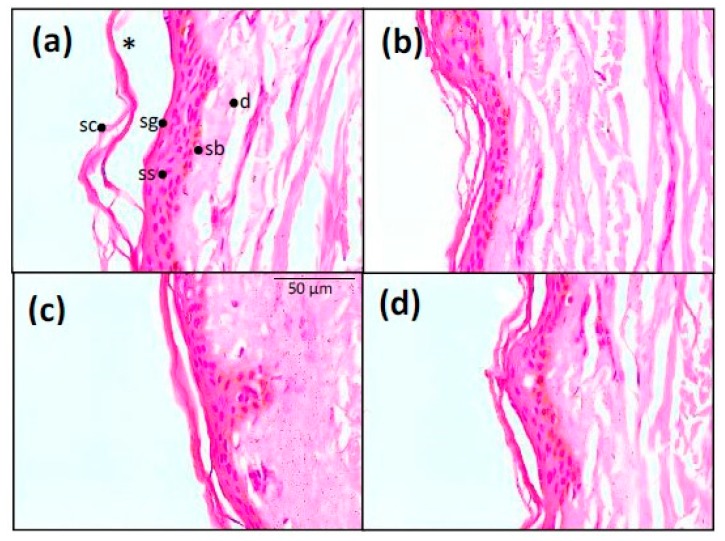
Histological analysis of skin structure. Hematoxylin and eosin staining of skin treated with: (**a**) BFZ-CF, (**b**) ME reference free of polysorbate, (**d**) ME reference with polysorbate, and (**c**) JMLP01BT (400× magnification). Sc = stratum corneum, sg = stratum granulosum, ss = stratum spinosum, sb = stratum basale, d = dermis, and * = loss of stratum corneum.

Epidermis treated with the commercial O/W cream (BFZ-CF) presented a slightly detached stratum corneum, followed by a normal stratum granulosum, stratum spinosum, stratum basale, and the dermis (Figure 9a). Skin treated with the MEs placebos, with or without polysorbate (Figure 9b,d), showed an intact stratum corneum with unaltered adjacent structures. The skin in presence of JMLP01BT (Figure 9c) showed a pattern similar to that of its respective ME placebo, with an intact stratum corneum, without abnormal histologic layers detachment. No sign of cellular alteration in stratum spinosum was observed.

### 3.7. Skin Integrity Assessment

**Figure 10:**
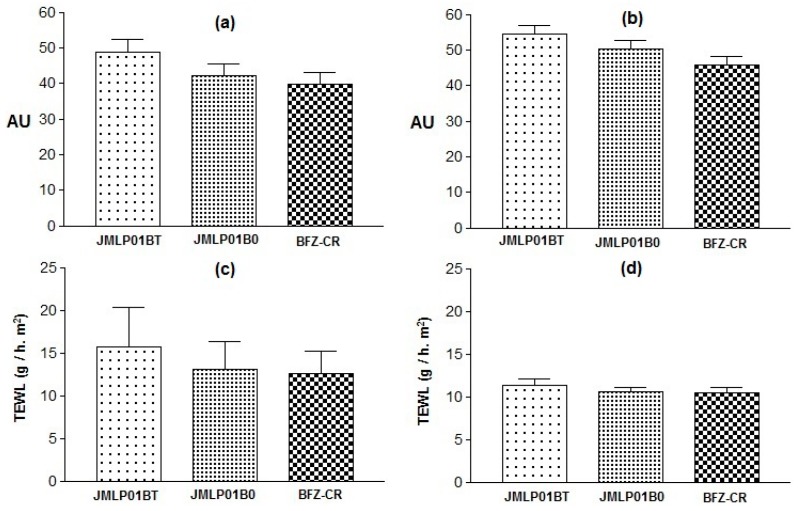
Stratum corneum Hydration (SCH) at *t*_0_ (**a**) and *t*_1_ (**b**), Transepidermal Water Loss (TEWL) at *t*_0_ (**c**) and at *t*_1_ (**d**). Variations are expressed as standard deviations.

The skin elasticity modified by JMLP01B0 was significantly lower than the values obtained with the other formulations (0.0521 ± 0.003 vs. 0.2232 ± 0.03 or 0.1605 ± 0.04 AU). Otherwise, no variations in Hydration or TEWL before (*t*_0_) and after (*t*_1_) forearm applications were demonstrated (see Figure 10 and Table 5). In fact, respective variations of TEWL (at the same skin temperature) for both MEs were similar.

## 4. Discussion

### 4.1. Physicochemical Properties and Stability

BFZ is practically insoluble in water (0.13 µg/mL, pH 7.4, 32 °C) and sparingly soluble in ethanol [33,34]. Its total dose in the MEs (10 mg/g) was fully dissolved in the lipophilic intermediate phase (17.5% of the total weight) [17,35,36]. The main component of this oil phase, LP, is a synthetic medium chain (C8-10) triacylglyceride which has better interfacial properties in comparison with classical oils. It is a good solvent for lipophilic active pharmaceutical ingredients and is associated with enhanced drug penetration. As described in several papers [37,38], LP stabilizes MEs either alone or in the presence of polysorbate 80. Such a mixture allows greater solubilization of hydrophobic model drugs, and since this oil phase limits the interaction between internal and external aqueous phases, it may influence the drug release pattern depending on the phase-distribution of the drug. As for the pH, the non-ionic surfactant increases slightly the pH of the MEs [39], does not react with ionic substances and has a good history of being safe in its use [4]. Considering the pKa of BFZ (pKa = 6.29) [40] and the pH of Tween^®^ 80 in aqueous solution (pH = 7) [39], a neutral pH in the aqueous phase is convenient to assure about a 50% ionization of drug favoring its transference from the oil phase to the external phase in contact with the skin. Sorbitan monooleate (grade 80) was selected a priori to achieve the best interaction with LP triglyceride in the oil phase and to intensify the subsequent BFZ penetration in stratum corneum.

As for the conductimetric results, the transference of NaCl from the inner layer W_1_ to W_2_ is not relevant due to the low conductivity values obtained at the end of the stability study [41]. It suggests the preservation of the phase’s distribution during ageing. An increase in conductivity of W_2_ would suggest the migration of electrolytes [42] as a result of diffusion or droplet breaking. In our case, the phase’s distribution of this ME is demonstrated as stable.

Droplet size analysis was carried out to detect possible changes in the distribution of the overall size and alterations in the volume mean diameter [43]. However, laser diffractometry only informs about the size distribution of the external globules of MEs [44], considering the dispersed volume fraction as being a simple O/W emulsion. This method, if it were combined with other ones, could give more information and an overview of the behavior of a multiple emulsion [45]. Assuming that BFZ is dissolved in the oil phase, this measurement of stability can be accepted as release indicating. No significant variations (α = 0.05) of droplet size have been detected during storage. Based on D[4,3] and D[3,2], the maintenance of space-fill, interfacial area, and thus, drug transport can be assumed [46]. Within experimental uncertainty both diameters in optical microscopy remained invariant over the whole storage period for each emulsion, indicating the absence of external and internal coalescence for samples kept at room temperature. The analyses of physical destabilization were measured using an optical analyzer Turbiscan^®^ Lab Expert to predict the instability phenomena within backscattering profiles by multiple light scattering. If the %∆BS profiles have a deviation of ≤ 2 %, it can be considered that there are no significant variations in droplet size. Variations ≥10% indicate instability risk [46]. The formulations in this study were milky and opaque, therefore only BS profiles were used to evaluate the physicochemical stability. As shown in Figure 3, no relevant variations of the droplet volume fraction (particle migration) or mean size (coalescence, flocculation) were observed on the MEs. Stability of the MEs can also be assumed to be larger than six months.

### 4.2. Rheological Properties

Rheological measurements have been used to assess the resulting skin application properties and also its stability [5,47,48]. As is shown in Figure 4a, the steady shear viscosity sharply decreases and there is an increase in shear rate from 0 to 100 s^−1^. The determination of the disturbance of the W_1_/O/W_2_ emulsion microstructure during the shearing process of rheological behaviour (Figure 4b) was evaluated by measuring the area of hysteresis loop (Pa/s) called “apparent thixotropy” [17,49,50]. The thixotropic area provides qualitative information about flow time dependence [51]. In fact, the shearing process could induce a phase inversion of the multiple emulsion to a W/O emulsion type in a semi-solid state and negative thixotropic patterns when they were observed. In the shearing conditions of the present study, the developed multiple emulsions showed a non-negative thixotropic pattern, indicating that the experimental conditions did not lead to phase-inverted emulsion. Nonetheless, this behavior cannot be described as real thixotropy since in multiple emulsions examined by a cone-and-plate viscometer, it is difficult to recover initial structure after being subjected to strong shearing”. In this sense, these flow curves (Figure 4b) indicate certain apparent thixotropy as the rheograms displayed a hysteresis loop.

The best kinetic model (Cross-kinetic equation) provides a general model for pseudoplastic materials and suggests non-Newtonian shear-thinning flow under shear. It demonstrates a good extensibility and spreadability on the skin surface for the MEs. El-Hadidy et al. [40] obtained similar pseudoplasticity of microemulsions containing polysorbate 80. In our case, the presence of polysorbate 80 in the ME has significantly increased viscosity and apparent thixotropy, which are profitable effects in that they increase the physical retention of the formulation [52]. This fact, in line with the effect of carbomer, modulates the consistency of the formulations working towards an optimal application over large skin areas, as other authors have shown [53]. Conversely, the commercial formulation exhibits a greater viscosity (Table 2, Figure 5) requiring a greater apparent thixotropic effect for a proper extension on skin.

The oscillatory sweep stress and frequency sweep tests have been used, basically, to find the storage modulus (G’) that measures the ability of the system to store recoverable elastic energy, and the loss modulus (G’’) that reveals the dynamic viscosity associated with unrecoverable viscosity loss. As shown in Figure 6 and Table 2, all formulations revealed a prevalence of the elastic over the viscous behavior (G’ > G’’). Jiao et al. [54] described these same properties in their formulations containing Tween^®^ 80, remarking its fluidification, as observed with JMLP01B0 and JMLP01BT. It is presumed that this elasticity favors the structural stability and resistance to external forces for longer periods of time [55].

### 4.3. Release and Skin Permeation

The experimental setup satisfied the required experimental standards as follows: spectrophotometric and chromatographic quantifications of BFZ were previously validated with acceptable linearity, precision, accuracy and recovery. Sink conditions in diffusion cells were guaranteed in all cases [56,57]. Mesh size of nylon membranes was clearly lower than droplet sizes, so one can be assured that the observed profiles are only due to the released drug and skin integrity before experiments [58] was assessed with TEWL measurements.

All release profiles have been described with the Higuchi function (square root), suggesting a depletion-limited release process and reaching asymptotic values rapidly after 5 h (Figure 7). It confirms that the oil intermediate phase acts as a drug reservoir-like with prolonged release towards the external aqueous phase and achieving a pseudo infinite-dose release model.

As initially enunciated, the objective of formulating a ME was the achievement of a lower skin permeation level and higher skin retention rather than a commercial O/W formulation. Skin permeation levels were *ca.* 10^−3^ times lower than the corresponding release levels. This difference is explained by the high lipophilicity of imidazolic drugs and their consequent low skin permeability. In fact, the formulation of drugs with limited permeability and solubility (BCS, class IV) offers a specific opportunity for a well-developed topical administration [59] due to their poor systemic absorption in animals and humans [47,48]. In our case, resultant values of BFZ permeation flux (Table 3) are slightly lower than those obtained by Hashiguchi et al. [60] with a BFZ lipophilic suspension through mouse skin (0.3386 µg/cm^2^/h)).

Currently, the treatment of *Tinea versicolor* and dermatophytosis [61] with Bifonazole requires its application once every 24 h for at least three weeks [1,2,62,63,64]. Given that these fungi commonly affect the epidermal cornified layers and the dermis [1], the intradermal retention of bifonazole appears as an indicator of its efficient delivery [62]. It is known that imidazolic drugs are prone to reversible bind to queratin in stratum corneum in direct relationship with their lipophilicity [60] prolonging its efficacy. Additionally, the presence of high concentrations of drug with specific surfactants in the formulation contributes to enhance the antimycotic effect [65]. In our case, drug accumulation levels in epidermis achieved with both MEs (Table 3) are higher than those with the commercial cream (0.4 µg/cm^2^), suggesting the possibility of lengthening the currently used 24 h administration interval up to an advantageous 48 h interval.

Even in the lower layers of the epidermis, the BFZ accumulated is several times higher than the in vitro minimum inhibitory concentrations for dermatophytes, especially along the hair follicles as the preferred route of penetration [66]. This marked intracutaneous distribution has been described, for example, with tioconazole in guinea pig skin, detecting (after 2 h) around 300 µg/g in stratum corneum, but only 25 µg/g in epidermis [67]. These results confirm that if BFZ is relicted in the lipophilic skin regions then its apparent permeation through the dermis can be reduced dramatically [68,69] favoring its antifungal activity in the epidermic tissue.

The efficiency of the drug release profiles from the MEs (cfr.: Equation 2) is highest for the polysorbate-containing formulation (JMLP01BT) and minimum for the commercial formulation (BFZ-CF), which is also directly proportional to the respective skin accumulation levels.

The drug penetration levels from the ME without polysorbate 80 (2.9 µg/cm^2^) are similar to those obtained by other authors [60] with a BFZ suspension (2.4 µg/cm^2^) in mouse skin. As for the polysorbate ME formulation, the skin accumulation levels are statistically higher (16.60 µg/cm^2^) than all the others (Table 3). This formulation dramatically increased the accumulation of BFZ inside the epidermic layers (400 µm thickness). Other authors [70], who directly related the amounts of drug in stratum corneum with the respective uptake of the excipients, have described a similar effect. In fact, the similarity among aliphatic molecules is sometimes associated with a potentiation of the enhancer activities [71]. For example, the effect of polysorbate 80 over the permeation of lipophilic drugs (logP above 4.15) through rat mucosal membranes [72] is related to an increase in diffusion coefficient, and thus, the drug transport, similarly with econazole [16]. A significant increase in skin deposition means improved cutaneous drug availability, and thus, the feasibility of an enlarged interval of administration.

### 4.4. Histological Analyses

While polysorbate 80 can cause anaphylactic reactions (non-immunological) in individuals prone to them, sensitization in selected patients with contact dermatitis is quite rare [73,74]. As a preliminary test for in vivo evaluation, residual skin samples of the in vitro tests were stained and observed in order to for any eventual histological alteration due to this substance. The representative microscopical images (Figure 9) illustrate that neither the presence of polysorbate alters the histological skin structure (placebo with vs. placebo without) and neither does the drug (Figure 9b,c). A for the other the O/W emulsion, it seems that the stratum corneum treated with the commercial emulsion clearly becomes detached from the viable epidermis.

### 4.5. Skin Integrity

In vivo results of stratum corneum hydration and transepidermal water permeability suggest a tendency to increase hydration and to decrease TEWL Figure 10, Table 4), probably due to an occlusive effect of the ME. This humectant effect is used [75] as an indicator of skin healing in antimycotic treatments [76]. In addition, TEWL is the best indicator of skin integrity [77] and similar effects are observed in both MEs. Thus, the adverse effects of polysorbate 80 over the skin integrity can be considered as negligible.

Regarding elasticity variations (*t*_1_–*t*_0_), significancy (*P* < 0.05) was observed between the effect of JMLPB0 and the polysorbate-containing ME and the commercial formulation (Table 4). This increase can be considered as non-irritancy relevant because it is not univocally attributable to the presence of polysorbate 80.

In aggregate, the combination of amphoteric and non-ionic surfactants in the ME has not resulted in a substantial alteration of skin integrity. Polysorbates are particularly useful to solubilize hydrophobic organic compounds enhancing its transit through porous substrates [78]. Its use in the formulation of a multiple emulsion has optimized the delivery of BFZ, a highly lipophilic drug, to the epidermic stratum corneum.

## 5. Conclusions

Bifonazole multiple emulsion with polysorbate achieves a clearly lower skin permeation and a higher skin retention than the conventional cream formulation. The formulation displayed an increase in pH, viscosity and viscoelasticity and a decrease of spreadibility constituting an interesting alternative for dermal application. The polysorbate-containing multiple emulsion has efficiently enhanced the ex vivo penetration of BFZ without altering the in vivo skin integrity and, thus, it optimizes the delivery of bifonazole, a highly lipophilic drug, towards the epidermic stratum corneum.

It has been shown that this type of formulation is likely to be tolerable in terms of biophysical skin-integrity and in terms of histopathological signals. In accordance with this, the proposed antifungal multiple emulsion is the preferred choice for the epidermal delivery of bifonazole with a prolonged interval administration and it is a promising alternative formulation for this imidazolic drug.

## Figures and Tables

**Table 1:** Formulation and percentage composition (*w*/*w*) of BFZ MEs without polysorbate (“–B0”) and with polysorbate, suffixed with “–BT”.

Components	Percentage Composition (*w*/*w*)	
	JMLP01B0	JMLP01BT
Oil phase (O)		
Bifonazole	1.00	1.00
Capric/caprylic triglyceride (Labrafac^®^ Lipophile 1349)	11.00	11.00
Cetyl palmitate	2.00	2.00
Cetyl dimethicone copolyol (Abil^®^ EM 90)	1.50	1.50
Sorbitan stearate (Span^®^ 60)	2.00	2.00
Internal aqueous phase (W_1_)		
Sodium chloride	0.25	0.25
Purified water at pH 6.6	32.25	32.25
External aqueous phase (W_2_)		
Carbomer (Tego^®^ Carbomer 341)	0.20	0.20
Cocamidopropyl betaine (Tego^®^ Betaine F)	0.70	0.70
Polysorbate 80 (Tween^®^ 80)	-	1.00
Purified water at pH 6.6	49.10	48.10

**Table 2:** pH, conductivity and particle size parameters by laser diffraction, for JMLP01B0, JMLP01BT and BFZ-CF at 24 h (first lines) and 6 months (second lines) (mean ± SD, *n* = 3).

Sample	pH	Conductivity (μm/s^−1^)	Droplet Size Analysis (μm)				
			D[4,3]	D[3,2]	D[*v*,0.1]	D[*v*,0.5]	D[*v*,0.9]
JMLP01B0	5.80 ± 0.04 6.01 ± 0.01	309.5 ± 6.5 336.3 ± 7.1	65.2 ± 0.8 75.2 ± 0.7	9.7 ± 0.2 10.5 ± 0.7	3.4 ± 0.3 4.1 ± 0.7	35.8 ± 0.3 33.8 ± 0.5	172.9 ± 6.3 189.2 ± 7.1
JMLP01BT	6.11 ± 0.02 6.30 ± 0.04	257.3 ± 3.3 289.0 ± 3.5	68.5 ± 1.0 60.8 ± 0.9	12.8 ± 0.1 12.1 ± 0.8	6.0 ± 0.2 6.3 ± 0.8	40.4 ± 5.6 49.5 ± 1.8	168.1 ± 3.4 165.02 ± 4.1
BFZ-CF	6.09 ± 0.04 N.A.	199.1 ± 6.3 N.A.	29.8 ± 9.8 N.A.	5.8 ± 0.5 N.A.	1.8 ± 0.2 N.A.	18.0 ± 2.1 N.A.	59.0 ± 13.9 N.A.

(N.A. Not applies).

**Table 3:** Mean viscosity values (mPa.s) at 10 s^−1^ and 100 s^−1^ (mean ± SD, *n* = 3) and oscillatory test of storage modulus (G’), loss modulus (G’’), and complex viscosity (η*) at 0.01 s^−1^ and 10 s^−1^.

Formulation	Frequency (s^−^^1^)	Viscosity (mPa.s)		Oscillatory Measurements (at 24 h)		
		at 24 h	180 days	G’(Pa)	G’’(Pa)	η*(Pa.s)
JMLP01B0	0.01	N.A.	N.A.	62.08	19.47	1035.00
	10	1745.0 ± 16.7	1705.0 ± 15.7	85.09	27.45	1.42
	100	237.5 ± 4.7	205.8 ± 2.6	N.A.	N.A.	N.A.
JMLP01BT	0.01	N.A.	N.A.	81.99	30.29	1391
	10	2176.0 ± 24.6	2093.5 ± 19.9	167.60	60.97	2.84
	100	295.8 ± 6.8	261.2 ± 4.1	N.A.	N.A.	N.A.
BFZ-CF	0.01	N.A.	N.A.	3736	1875	66,530
	10	19,301.7 ± 99.7	19,240.5 ± 116.9	15,940	4160	262.20
	100	1901.3 ± 30.9	1963.0 ± 23.7	N.A.	N.A.	N.A.

N.A.: Not Apply.

**Table 4:** Drug release parameters (K and efficiency), skin permeation flux and skin penetration of BFZ from both MEs and the commercial formulation (mean± SD). Statistical differences are indicated with superscript letters.

Formulation	K Higuchi	AUC_0_^5^/(Q5·T)	Permeation Flux	Skin Penetration	
	(µg·h^−1/2^)	(h)	(µg/cm^2^h)	(µg/g)	(µg/cm^2^)
JMLP01B0	539.3 ± 71.5	0.644 ± 0.072	0.137 ± 0.008 ^b^	13.12 ± 1.24 ^b^	2.87 ± 0.36
JMLP01BT	690.4 ± 97.8 ^a^	0.716 ± 0.026	0.064 ± 0.012	106.20 ± 8.73	16.60 ± 7.09
BFZ-CF	780.0 ± 87.5	0.692 ± 0.098	0.132 ± 0.009 ^b^	4.91 ± 0.72 ^b^	0.38 ± 0.07 ^b^

^a^ Statistical differences with JMLP01B0. ^b^ Statistical differences with JMLP01BT.

**Table 5:** Differences (*t*_1_–*t*_0_) of in vivo skin integrity tests with JMLP01B0, JMLP01BT, and BFZ-CF (mean ± SD, *n* = 10). Statistical differences between formulations are indicated with superscripts.

Formulations	Skin Elasticity Mean Differences (AU)	Skin Hydration Mean Differences (AU)	TEWL Mean Differences (g/h·m^2^)
JMLP01B0	0.0521 ± 0.003 *	8.1 ± 7.8	3.6 ± 1.4
JMLP01BT	0.2232 ± 0.03	3.6 ± 1.4	3.6 ± 1.9
BFZ-CF	0.1605 ± 0.04	6.0 ± 6.5	1.5 ± 2.3

AU: Arbitrary units. * Statistical differences with JMLP01BT and BFZ-CF.
